# Population Viability and Vital Rate Sensitivity of an Endangered Avian Cooperative Breeder, the White-Breasted Thrasher (*Ramphocinclus brachyurus*)

**DOI:** 10.1371/journal.pone.0148928

**Published:** 2016-02-09

**Authors:** Jennifer L. Mortensen, J. Michael Reed

**Affiliations:** Department of Biology, Tufts University, Medford, Massachusetts, United States of America; University of Lleida, SPAIN

## Abstract

Social behaviors can significantly affect population viability, and some behaviors might reduce extinction risk. We used population viability analysis to evaluate effects of past and proposed habitat loss on the White-breasted Thrasher (*Ramphocinclus brachyurus*), a cooperatively breeding songbird with a global population size of <2000 individuals. We used an individual-based approach to build the first demographic population projection model for this endangered species, parameterizing the model with data from eight years of field study before and after habitat loss within the stronghold of the species’ distribution. The recent habitat loss resulted in an approximately 18% predicted decline in population size; this estimate was mirrored by a separate assessment using occupancy data. When mortality rates remained close to the pre-habitat loss estimate, quasi-extinction probability was low under extant habitat area, but increased with habitat loss expected after current plans for resort construction are completed. Post-habitat loss mortality rate estimates were too high for projected populations to persist. Vital rate sensitivity analyses indicated that population growth rate and population persistence were most sensitive to juvenile mortality. However, observed values for adult mortality were closest to the threshold value above which populations would crash. Adult mortality, already relatively low, may have the least capacity to change compared to other vital rates, whereas juvenile mortality may have the most capacity for improvement. Results suggest that improving mortality estimates and determining the cause(s) of juvenile mortality should be research priorities. Despite predictions that aspects of cooperative systems may result in variation in reproduction or juvenile mortality being the most sensitive vital rates, adult mortality was the most sensitive in half of the demographic models of other avian cooperative breeders. Interestingly, vital rate sensitivity differed by model type. However, studies that explicitly modeled the species’ cooperative breeding system found reproduction to be the most sensitive rate.

## Introduction

Understanding the drivers of population decline and the dynamics of the extinction process are interesting research problems (e.g., [1–3]), with important applications to species management [4, 5]. A common tool used to investigate the effects of these drivers is demographic modeling [6]; the most common approaches have been matrix models (e.g., [7]) and individual-based models (e.g., [8]), but new approaches are being explored, such as multi-agent systems modeling (e.g., P systems models [9]). When these models are used to understand, predict, and manage extinction risk of small or declining populations, they are often called population viability analysis (PVA) [10]. In particular, PVAs are used for assessing risk category for the International Union for Conservation of Nature (IUCN) Red List of Threatened species [11], in guiding funding decisions (e.g., [12]), and in planning species’ recovery (e.g., [13])–and PVAs have been employed in over 140 publications from 2000–2014 (Web of Science, 2014, search terms included *PVA*, *population viability analysis*, *extinction risk model*, *viability model*). Consequently, improving the structure and accuracy of population projection models is a priority [14].

One potential way to improve PVAs is to incorporate species behaviors that might affect population persistence [15–18]. Behaviors explicitly modeled in PVAs have included dispersal [19], habitat selection [20], and Allee effects [21]. In contrast to Allee effects, which decrease persistence of small populations [22], social behaviors that might buffer populations against extinction have received little attention [16, 23, 24]. One type of social behavior, cooperative breeding, might buffer extinction risk through a variety of mechanisms. Cooperative breeding has been reported in 5–6.5% of bird species [25, 26]. It encompasses a diverse group of social systems, but typically involves individuals (helpers) helping to care for young that are not their own offspring [25]. In facultative systems, as opposed to obligate systems, helpers are not necessary for a breeding pair to raise young to independence.

It has been suggested that cooperative breeding might buffer extinction risk. For example, after a disease wiped out 45% of breeding Florida Scrub-Jays (*Aphelocoma coerulescens*), all vacant breeding positions were filled within two years by birds previously acting as helpers [27]. Similar buffering occurred in the cooperatively breeding Galápagos Mockingbird (*Mimus parvulus*) after sequential climatically harsh years [28]. These observations have been reinforced by modeling studies of cooperative breeder population dynamics that showed that very small populations, when aggregated, were resilient to reductions in population size because helpers are a pool of potential breeders [19, 29]. Other potential mechanisms by which cooperative breeding might reduce extinction risk include increasing reproduction and decreasing variance in reproduction [30], increasing nestling and breeder survival via enhanced resource or predator defense [31], and expanding local carrying capacity [32] and minimizing density dependent feedback when adult numbers increase. Consequently, incorporating the effects of cooperative breeding into PVAs, for appropriate species, could be important. Here we present a PVA of an endangered cooperatively breeding bird, the White-breasted Thrasher (*Ramphocinclus brachyurus*), at the stronghold of its distribution, where it recently lost a significant amount of habitat.

**Fig 1:**
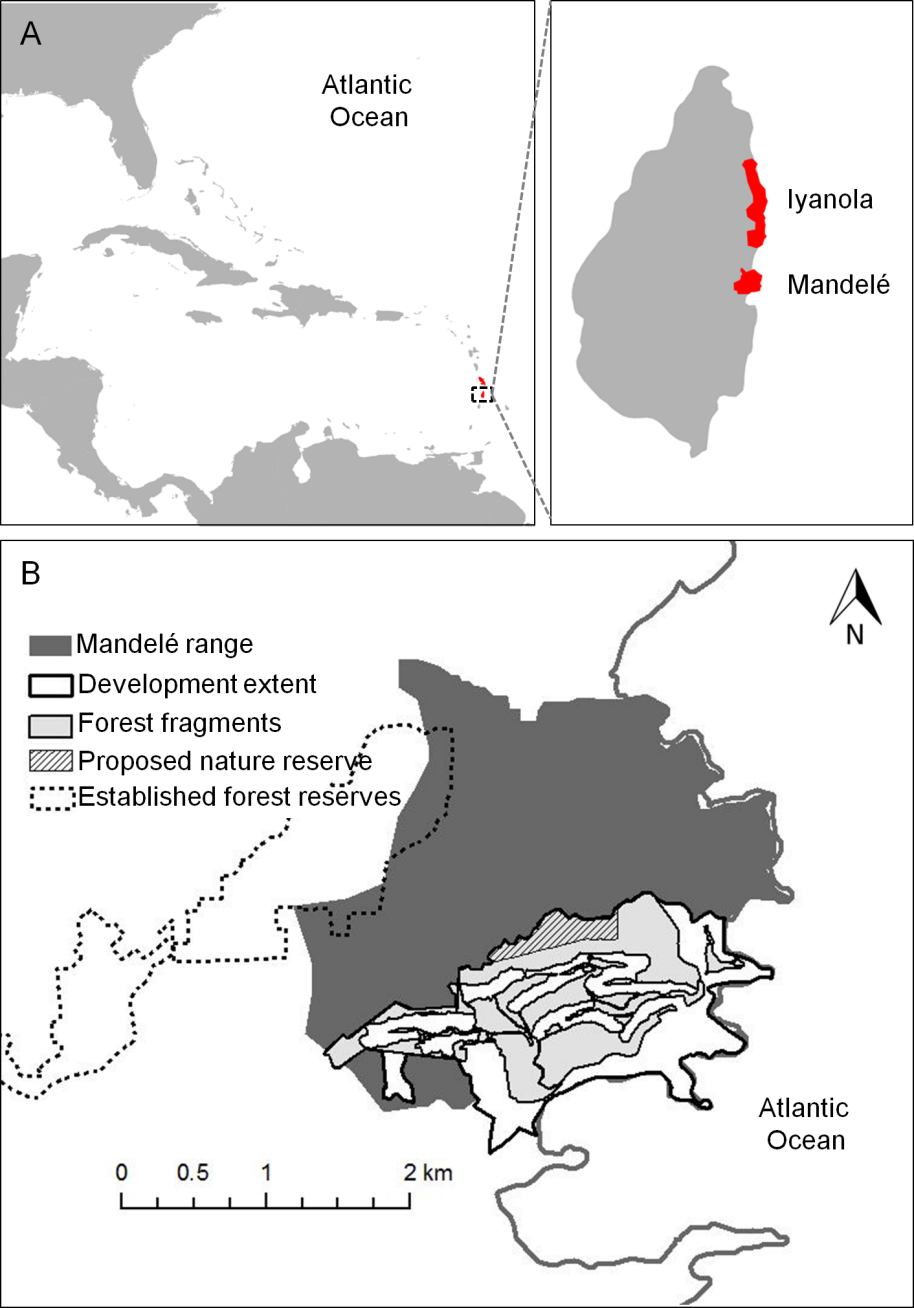
Geographic distribution of the White-breasted Thrasher. (A) White-breasted Thrashers are restricted to the islands of Saint Lucia and Martinique, shown in red. Data source: CIA World DataBank II. (B) Approximately 80% of the species’ global population inhabits the central east coast of Saint Lucia within the Mandelé range. This range is a mix of dry forest habitat and human land use. Twenty percent of habitat within the range was lost due to resort construction beginning in 2005; the approximate area of forest remaining on the development property is shown in light gray. Only 4% of the Mandelé range overlaps with federal forest reserves. Data source: Recreated and modified from [33, 34].

The White-breasted Thrasher is a facultative cooperative breeder [33, 34] that is endemic to the islands of Saint Lucia and Martinique (Fig 1; [35]). The species has a global population size of fewer than 2000 adults, 15% of which live on Martinique’s Caravelle Peninsula. The remaining 85% live on the east coast of Saint Lucia in two ranges–Iyanola and Mandelé–that are separated by approximately 4 km [34, 36]. The Mandelé range is the largest remaining habitat for this species; it contains about 80% of the global population of White-breasted Thrashers and 92% of the Saint Lucian subspecies. Development of a tourist resort began in the Mandelé range in 2005, and to date, 20% of the species’ habitat within the range has been destroyed, constituting 16% loss of habitat in Saint Lucia (Fig 1; [37]). The development site now consists of habitat fragments surrounded by contiguous forest. The Mandelé population, and in particular the area in and around the construction site, is the source of demographic data for the model presented here. Our goals in this paper were to (1) develop a PVA for the White-breasted Thrasher under extant conditions, and (2) to compare estimated current extinction risk to risks prior to the recent habitat loss and to possible future habitat loss. (3) Our final set of goals was to use sensitivity analyses to (a) determine the parameters that most influence persistence and population growth, (b) identify where more accurate data would improve viability predictions in order to guide future research efforts, and to (c) compare vital rate sensitivities to those reported for other avian cooperative breeders.

## Materials and Methods

### Study area: the Mandelé range, Saint Lucia

Our focal population inhabits the Mandelé range (Fig 1; 13.89°N, 60.89°W), an approximately 630 ha parcel [36] of littoral scrub, shrubland, deciduous seasonal forest, and semi-evergreen seasonal forest [38]. White-breasted Thrashers are deciduous forest specialists [34]. In the Mandelé range, the deciduous forest is largely secondary growth [34, 38], and is punctuated by gardens, pasture, clearings for charcoal production, and the 10-ha Bordelais Correctional Facility. Only 4% of the Mandelé range is federally protected land; the remaining area is either privately held or unprotected Crown land [36]. The range lies within the larger Mandelé Important Bird Area (826 ha; [39]).

Our study relied on data gleaned from the literature, as well as unpublished field data. All data necessary to recreate this PVA can be found within the text. Collection of our field data was approved by Tufts University’s Institutional Animal Care and Use Committee (M2009-66 A-1, M2013-43). Field study areas were private property, and were accessed with permission from the Saint Lucia Forestry Department and the Managing Director of DCG Properties Ltd.

### White-breasted Thrasher individual-based model

We used an individual-based stochastic simulation modeling package, VORTEX [40, 41] versions 9.99 [42] and 10.0 [43], to evaluate viability of the White-breasted Thrasher population in the Mandelé range. We chose to use build an individual-based model because it allowed us to include the information about the species’ social system that we thought was important. While a Lefkovitch model has been proposed as a simpler, alternative approach to include social behavior in population modeling [44], we do not have information on annual transition probabilities between some stages (e.g. between helper and breeder), so a stage-based model built for our study species would lack that social behavior. Our primary time frame for evaluating population viability was 40 generations (200 years for this species; as recommended by [45, 46]). Because the IUCN uses a standard time frame of 100 years when using PVAs to assess Red List status, however, we also ran a subset of scenarios using that target time as suggested by [14].

We created our baseline model at the extant carrying capacity (referred to as 1K), which corresponds to the amount of White-breasted Thrasher habitat available in the Mandelé range after recent habitat loss, and is described further below. We parameterized the model using demographic data from our own field studies of the White-breasted Thrasher in the Mandelé range and from data reported in the literature (Table 1). We ran 1000 iterations for each scenario. For all scenarios we recorded: (1) the probability of extinction at 200 years; (2) the distribution and median time to extinction for populations that went extinct; (3) the distribution and mean population size (± SD) of extant populations at 200 years; and (4) the deterministic and stochastic population growth rates (± SD). VORTEX calculates the deterministic growth rate using life table analysis and the Euler-Lotka equation, while the stochastic growth rate is calculated from the natural log of lambda [47]. We defined a population to be extinct when only one sex remained. We did not perform any statistical analyses comparing results of alternative scenarios because p-values are determined in part by sample size so we could find statistical significance merely by increasing the number of iterations. Here we review data used to parameterize the PVA as well as model assumptions.

**Table 1:** Parameters used to build individual-based models for the White-breasted Thrasher population viability analysis.

			Range explored by sensitivity analysis methods		
Parameter	Baseline value	Source	Perturbation	Conventional	Logistic regression^e^
*Reproduction*					
Breeding system	Monogamy	[34]	-	-	-
Minimum age of reproduction (years)	2	[33, 34]	-	-	-
Maximum age of reproduction (years)	10	-	-	-	-
Max. # successful clutches per breeding female per year^a^	2	[33, 34]	-	-	-
Distribution of successful clutches per breeding female per year (0:1:2 clutches; ‘breeding success’)	0.27:0.54:0.19	[33, 34]	0–1, by 0.1	± 10%	0.43:0.42:0.15–0.17:0.62:0.21
Max. # of fledglings per successful clutch	2	[33, 34]	-	-	
Distribution of fledglings (1:2 fledglings)	0.34:0.66	[33, 34]	-	-	0.43:0.57–0.15:0.85
Sex ratio at birth (in % males)	0.50	[34]	-	-	-
Females breeding ± EV^b^	Eq 1 ± 0.06	[33, 34]	Ceiling model, Eq 2	± 10% (EV: ± 10%)	(EV: 0.03–0.12)
Males in breeding pool	Eq 1	[33, 34]	Ceiling model, Eq 2	-	-
*Annual mortality*^c^					
Juvenile mortality (fledge-age 1) ± EV	0.49 ± 0.20	[34]	0–1, by 0.1	± 10% (EV: ± 10%)	0.34–0.68 (EV: 0.09–0.22)
Adult mortality (age 1+) ± EV	0.13 ± 0.10	[34]	0–1, by 0.1	± 10% (EV: ± 10%)	0.13–0.26 (EV: 0.09–0.22)
*Population parameters*					
Initial population size	1012	[33, 34, 36]	-	-	-
Carrying capacity ± EV	1085 (1K^d^) ± 3%	[33, 34, 36]	1319 (1.2K), 869 (0.8K), 191 (0.17K)	± 10%	100–2000

^a^ A clutch is defined as successful if ≥ 1 chick fledges.

^b^ Standard deviation is used to represent environmental variation (EV) in VORTEX.

^c^ The adult rate is apparent mortality estimated using Program MARK [48]. Due to ambiguity in methods used to estimate juvenile mortality [34], the value here is re-sighting rate calculated from published data and has not been adjusted for detection probability.

^d^ 1K corresponds to actual post-habitat loss forest cover; see text for other cover values.

^e^ Input values used in this sensitivity analysis come from distributions of the parameter ranges shown here.

Our model, which begins tracking individuals at fledging, includes three age classes: hatch-year (fledgling to age 1), second-year (age 1 to 2), and after-second year (age 2+). Transitions between age classes were determined by mortality rates of hatch-year birds (hereafter, juvenile) and after-hatch year birds (age 1+; hereafter, adult) [34]. Capture data [33, 34] suggest that breeders are always after-second year birds; age 2 was used as a minimum age of reproduction in the model. Thrasher longevity is not well documented, although we have recaptured or resighted banded individuals that were at least ten years old; age 10 was used as a maximum age of reproduction in the model (Mortensen unpubl. data). There is no evidence of reproductive senescence, so we assumed that reproductive output remains constant with age. Mean clutch size, calculated for nests found before or during laying, was 1.97 ± 0.03 eggs before habitat loss (n = 96 nests; [34]) and 1.97 ± 0.03 eggs after habitat loss (n = 31 nests; [33]), and we assumed a 1:1 sex ratio at hatching. We defined a clutch as successful if it fledged ≥ 1 chick. While thrashers can lay up to four clutches over the course of a breeding season [34], usually there are no more than two successful clutches within a year. Only once–out of 85 total territories monitored in three studies spanning ten years ([33, 34], Mortensen unpubl. data)–did a female raise three successful clutches during a season [34]. We used breeding success data from these 85 territories to determine the distribution of clutch successes per female during the breeding season, and data from 166 nests to determine the distribution of chicks fledged per successful clutch ([33, 34], Mortensen unpubl. data). We assumed that there were no effects of inbreeding in this population, or alternatively, that effects are already represented in parameter estimates [49].

White-breasted Thrashers are facultative cooperative breeders [32, 33]. Groups are composed of a breeding pair and 0–4 helpers. Breeding pairs are socially and genetically monogamous, and helpers, in a near 1:1 sex ratio, are predominantly retained offspring of the breeders [32, 50]. Mean group size ± SE was 2.4 ± 0.07 adults before the habitat loss described above [34] and 2.8 ± 0.08 adults after [33]; 34% and 63% of territories contained cooperative groups before and after habitat loss, respectively. We did not model thrasher breeding success as a function of group size; while there is some evidence that cooperative groups have higher reproductive output than do lone pairs [34], the generalization of this pattern is unknown and having more helpers does not seem to affect reproductive success.

We modeled percent females attempting to breed as a function of number of territories, as

$$P(breeding\;females)=\{\begin{array}{c} \;1\;\frac{\#\;territories}{\#\;adult\;females}\geq 1\\ \frac{\#\;territories}{\#\;adult\;females}\;\frac{\#\;territories}{\#\;adult\;females}<1 \end{array}$$ (1)

The percent of males in the breeding pool was calculated in a similar way, with # adult females replaced with # adult males in the denominator. Cooperative breeding was modeled implicitly in this function. When modeling percent breeding, we assumed that every individual capable of breeding tried to breed when there were more available territory sites than potential breeders. We also assumed that there were no floaters. Percent of adults breeding varies with number of territories, but also with fluctuations in group sizes–i.e. after all territories are occupied, group sizes can still increase. We used five years of group size data to estimate environmental variation in percent females breeding [33, 34]. Specifically, we assumed a 1:1 adult sex ratio and one female breeder per group to calculate variance in percent breeding across time. We assumed that there was no density-dependent effect on reproductive output. Finally, we assumed that environmental variation was concordant across reproduction and survival, i.e. bad years for reproduction were also bad years for survival. While we do not have data to know if environmental variation affects reproduction and survival simultaneously or independently, we think that in general, the factors affecting chick production are the same as those affecting mortality, though the strengths of the effect may be different.

We determined the maximum number of territories possible in the Mandelé range by multiplying total range area by territory density. Territory density estimated from territory mapping was 0.71 territories ha^-1^ [34]. This might be a conservative estimate of territory size because the study area contained pockets of non-habitat. Using an estimate of population density from distance sampling methods [36], we calculated an average territory density of 0.67 territories ha^-1^, an estimate similar to that generated by the territory mapping method [34]. Consequently, we used a value of 0.70 territories ha^-1^. We assumed that territory size was fixed over time. We also assumed that habitat quality was homogenous across the Mandelé range, that there were no edge effects on reproductive success due to habitat fragmentation, and that all habitable forest was occupied by thrashers. We think the last is a reasonable assumption, given that this species is a facultative cooperative breeder and that nest searching and territory monitoring revealed no large tracts of apparently suitable yet unoccupied habitat [33, 34].

We used the estimate of territory density (as described above) in our calculations of initial population size and carrying capacity. We calculated initial population size as number of territories multiplied by average group size based on field data collected before and after habitat loss; we set group size at 2.4 adults in the before habitat loss scenarios [34] and at 2.8 adults in the after habitat loss scenarios [33]. We do not have data on the precise age-class distribution of the population, so we assumed a stable age distribution. We calculated carrying capacity for all scenarios in the same way; we multiplied number of territories by the most common cooperative group size, which was three adults [33, 34], assuming that this represented a saturated population. In the White-breasted Thrasher system, variation in carrying capacity is likely due to creation of territories by budding and elimination by territory merging or habitat loss. We calculated environmental variation in carrying capacity using territory creation data to calculate mean percent change in annual number of territories [34].

White-breasted Thrashers live in areas at risk of hurricane strikes–their entire population resides < 2 km from the east coasts of two Lesser Antillean islands–and their breeding season [34] largely coincides with the Northern Atlantic hurricane season. Five hurricanes made landfall on Saint Lucia between 1960 and 2010 during the thrasher breeding season [51]. We do not know the impact of these hurricanes on thrasher survival and reproduction. However, we suspect that direct adult mortality is low [52] and that there is little impact on abundance, as was the short-term pattern for several other Caribbean forest insectivores after Hurricane Hugo impacted the region in 1989 [53–55]. The reproductive response of avian populations following hurricanes is variable; some species have delayed breeding seasons, some do not, and some do not breed at all [52]. Because thrashers are multi-brooded and because they do not have narrow nest site requirements [34], we do not expect a hurricane to cause total reproductive failure for that year. Consequently, all of the PVA scenarios included hurricanes with an annual probability of 0.1 that resulted in no additional mortality but a 50% reduction in annual breeding success.

Evidence to date suggests that the three populations of thrashers are completely isolated: of 693 birds banded in the Mandelé range since 2002, none have been reported from the other populations [37], and mean reported dispersal distances are < 200 m [32, 33]. Low levels of dispersal might nonetheless occur, so we investigated the effects of 1 and 5 female juvenile immigrants per year on persistence metrics.

### Model sensitivity

We examined the sensitivity of White-breasted Thrasher population dynamics to shifts in vital rates in three ways: (1) perturbation analysis, (2) relative sensitivity, and (3) a logistic regression approach. Each approach provides slightly different information; perturbation analysis shows which parameters are closest to their population-crash values, relative sensitivity assesses how small changes in vital rates affect population growth, and the logistic regression approach is used to determine which parameter explains the most variability in probability of extinction [56]. We also examined sensitivity of viability metrics to our chosen population growth model.

For the perturbation analysis of vital rates, we varied juvenile mortality, adult mortality, and the distribution of number of successful clutches per breeding female per year. For the last vital rate (hereafter referred to as breeding success), we varied the proportion of breeding females producing 0 successful clutches while keeping the ratio of those producing 1 and 2 successful clutches constant. Specifically, we varied the proportion of 0, 1, and 2 successful clutches per breeding female from 1:0:0 to 0:0.74:0.26. We focused on this aspect of reproduction because our data show it is much more variable than other parameters included in our model such as clutch size or number fledged per successful clutch. We varied the rates individually from 0–1, while holding the other parameters constant, in order to determine at what values persistence likelihood and population size dropped to a low level (as done by [57]). Each sensitivity scenario was run at the extant carrying capacity (1K) for 1000 iterations. In addition, we systematically changed adult mortality and breeding success at the same time to assess the rates required for population persistence across three time scales: 5, 20, and 200 years.

We performed a more conventional sensitivity method to assess sensitivity of population growth to small changes in White-breasted Thrasher parameter values. We did this by varying, one at a time, breeding success, percent females breeding, environmental variation in percent females breeding, carrying capacity, juvenile mortality, adult mortality, and environmental variation in mortality of both age classes ± 10% of their baseline value (Table 1). We used this method in order to compare our results to those published for other cooperative breeders. Each scenario was projected over 200 years and 1000 iterations. We calculated relative sensitivity of the model to changes in each parameter by (λ_+_—λ_−_)/(0.2*λ_0_), where λ_+_ and λ_−_ are the output from the adjusted parameter values, λ_0_ is the output of the baseline model, and 0.2 is the total perturbation of the parameter values (± 10%) [58]; because VORTEX begins each simulation with a stable age distribution, we were able to use the instantaneous growth rate output from VORTEX to calculate mean change in lambda. Parameters that show sensitivities > 1 or < -1 had a disproportionate effect on the population growth rate [58].

We also used a logistic regression approach [59] for a third method of sensitivity analysis. Unlike perturbation analysis or conventional sensitivity where, typically, one parameter is varied at a time, in the logistic regression method multiple parameters are varied simultaneously to more fully sample the parameter space and it is considered a global sensitivity analysis [60]. We treated breeding success a similar way as in the perturbation analysis described above, but here, the distribution of 0, 1, and 2 successful clutches was limited by 0.43:0.42:0.15 and 0.17:0.62:0.21. We used Latin Hypercube Sampling in VORTEX to create 1000 parameter sets with input values chosen from uniform distributions of empirically observed parameter ranges (Table 1). All simulations were run under baseline conditions (see Table 1 for values) with the exceptions of the specific parameters being varied. We ran 10 iterations of each parameter set, resulting in a dataset of 10,000 populations that were classified as extinct or extant at year 200. We analyzed the VORTEX simulation results using the car package [61] in R version 3.1.2 [62]. We checked the logistic regression assumption of a linear relationship between each of the predictors and the link function by visual inspection of partial residual plots, and found no substantial deviations from a linear trend. We evaluated the importance of the predictors by comparing their standardized regression coefficients (regression coefficient divided by its standard error; [59]).

Due to the White-breasted Thrasher cooperative social system, we assumed there would be little density-dependent pushback on percent of adults breeding until the population was very close to carrying capacity. We compared two alternative percent-breeding functions to our baseline function: a traditional logistic model and a ceiling-only model. For the traditional logistic model, we used the equation provided in VORTEX:

$$P(females\;breeding)=\left\{P\left(0\right)-\left[\left(P(0)-P(K)\right){\left(\frac{N}{K}\right)}^{B}\right]\right\}\frac{N}{N+A}$$ (2)

where P(0) is the proportion of females breeding at a low population size and P(K) is the proportion breeding at carrying capacity; 1.0 and 0.67, respectively. We calculated proportion breeding at carrying capacity by dividing the number of breeding females per group by the number of females per group, assuming an equal group sex ratio [34] and the most common cooperative group size, which was three adults [33, 34]. We did not include an Allee effect (A = 0) and examined one value of B (B = 2), a shape parameter that describes how far from carrying capacity density pushes back on reproductive output.

### Effects of habitat area

From the analyses described above, we found that White-breasted Thrasher population dynamics are sensitive to adult mortality rates. Consequently, we assessed the effect of habitat area on population dynamics over a range of adult mortality levels by co-varying carrying capacity from 0 to 1500 and adult mortality from 0 to 1. All other parameters remained at baseline settings. We used quasi-extinction (threshold at 100 individuals, which is about 20–30 breeding females in our system) rather than one-sex remaining as our definition of extinction in assessing habitat loss to be precautionary. A population size of 100 should feel strong effects of demographic stochasticity (chance events in reproduction and survival; [63]), and is much smaller than the minimum population size recommended for avoidance of inbreeding depression or maintenance of evolutionary potential [64]. We also evaluated extinction risk at four specific habitat area scenarios: (1) area available before habitat loss (628 ha, referred to as 1.22 K), (2) extant habitat area (517 ha, 1 K), (3) projected habitat remaining upon resort completion (414 ha, 0.80 K), and (4) extensive habitat loss expected from a completed resort–the first on the east coast of the island–catalyzing further tourist development in the area (91 ha, 0.17 K; corresponds to all habitat lost in the Mandelé range except for the proposed 14 ha resort nature reserve, Crown land, and the 4% of the range that is federally protected). We used an estimate of 628 ha in the Mandelé range for the before-habitat-loss scenario [36]. We calculated habitat area remaining for the three other scenarios, respectively, using habitat loss estimates from [33], development plans [36, 65], and cadastral and land use maps. We converted these areas to number of individuals for use in VORTEX using the same method described previously.

In addition to performing simulations of habitat loss, we used a minor modification of the relatively simple model presented in [66] to estimate, for comparative purposes, the expected reduction of population size after a change in area of occupancy:

$$c=1-\frac{\text{log}(1-p_{c})}{\text{log}(1-p)}\times b$$ (3)

Here *p* is the original occupancy of a species in a region, *p*_*c*_ is the occupancy after a proportion of the population is removed, and *c* is the resultant percent reduction in population size. Because this method assumes constant population density across periods, we included an additional parameter, *b*, to adjust for the White-breasted Thrasher social system, which allows density to differ between the time periods (group sizes on a territory increase). We parameterized the equation using White-breasted Thrasher occupancy data from the Mandelé range from 2006–2009 [67]. *p* and *p*_*c*_ are the proportion of 250 x 250 m cells occupied before (2006 occupancy data) and after (mean of 2007–2009 occupancy data) habitat loss, respectively. *b* is mean territory group size before [34] divided by group size after [33] habitat loss; in our model *b* = 0.87 [33, 34].

### Population viability analyses of avian cooperative breeders

We wanted to compare our results, particularly vital rate sensitivity, to those from other species of cooperative breeders. To do this, we searched Web of Science for published viability analyses and sensitivity analyses of cooperative breeding birds using the search terms *PVA*, *population viability*, *viability analysis*, *MVP*, *sensitivity analysis*, or *sensitivity testing*, and *bird* or *avian*. We verified cooperative breeding status of each species using the species list in [26] and included papers in our sample if a sensitivity analysis was performed. We did not include species described as occasional cooperative breeders. Our final list was not intended to be an exhaustive review, but a sample of published vital rate sensitivity analyses of cooperative breeder PVAs. We calculated an effect size for sensitivity analysis results in each paper by finding the proportional change in metric between the top two most sensitive parameters; we report the top two parameters if effect size between the two was less than 10%.

## Results

### White-breasted Thrasher individual-based model

Under baseline conditions, the White-breasted Thrasher population persisted over the 200-year time frame (40 generations; Table 2). The population was predicted to increase by an average of 9% per year, resulting in a mean final population size of 961 birds, although there was a wide range of possible results (range = 69–1182 birds, S1 Fig; deterministic r = 0.120). The simulation time period – 100 or 200 years–had little effect on any of the predicted viability measures. Similarly, our choice of population growth model had little effect on viability measures (Table 2). The baseline and ceiling models performed almost identically; the VORTEX density-dependent model predicted a stochastic growth rate of nearly half of the other two models, leading to a smaller mean population size at 200 years, but predictions for all three models were within 1 SD. Low and high (1 and 5 juveniles year^-1^, respectively) rates of immigration into the Mandelé population had minor effects on viability measures compared to the baseline model (Table 2).

**Table 2:** Comparison of White-breasted Thrasher population viability measures across scenarios.

Scenario	Probability of extinction	Stochastic population growth rate (r)^a^	Population size^a^
Baseline (200 years)	0	0.089 (0.203)	961 (169)
100 yrs	0	0.089 (0.203)	959 (165)
Ceiling growth model	0	0.102 (0.204)	969 (170)
VORTEX growth model	0	0.046 (0.199)	890 (201)
1 immigrant year^-1^	0	0.09 (0.203)	967 (169)
5 immigrants year^-1^	0	0.095 (0.203)	973 (160)

All models were run under baseline conditions (see Table 1 for values) with the exception of the specific parameter being varied.

^a^ Values are means (standard deviation). Means were calculated only from iterations that persisted over the entire time frame.

### Model sensitivity

**Fig 2:**
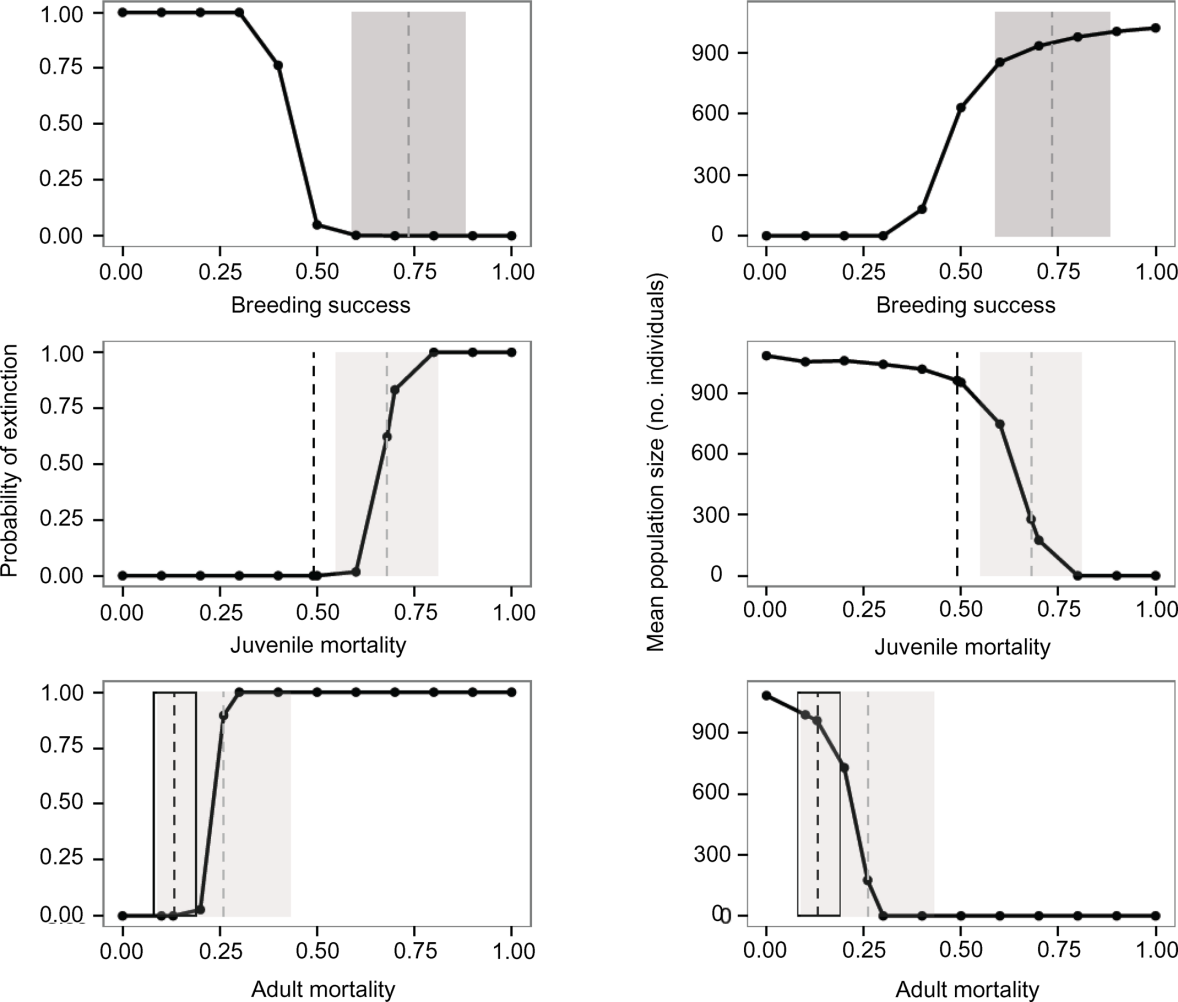
White-breasted Thrasher perturbation sensitivity analysis. Populations were projected at extant carrying capacity (1K) for 200 years. The hashed vertical lines and boxes show mean vital rates ± 1 SD before habitat loss (black outline; [34]), after habitat loss (light gray filled; [33]), and across both time periods (dark gray filled). Breeding success is the proportion of breeding females that produce at least one successful brood per season. Mean population size includes only the iterations that persisted over the entire time frame.

Perturbation analysis showed that breeding success could be reduced considerably from baseline levels (0.73; Fig 2) before effects on persistence metrics were notable. Breeding success had to fall below 0.5 (meaning that only half of breeding females produced at least one fledgling per breeding season) before population size began to fall and probably of extinction began to rise.

Population size and extinction probability were sensitive to increases in juvenile mortality (Fig 2). The threshold across which population persistence sharply declined occurred between 0.6 and 0.7. Juvenile mortality estimates calculated from field data before habitat loss (0.49; vertical dashed black line) allow for 100% population persistence, but the mean rate from after habitat loss does not (0.68; vertical dashed gray line ± SE). Minor increases in adult mortality led to substantial reductions in population viability and final population size (Fig 2). Probability of extinction quickly increased to 100% between adult mortality rates of 0.2 and 0.25. Empirically documented adult mortality before habitat loss (0.13; vertical dashed black line ± SE) was below this threshold value, but the mortality rate estimated after habitat loss was above it (0.26; vertical dashed gray line ± SE). Mean population size showed a similar threshold response to increasing adult mortality.

The effects of changing adult survival and breeding success at the same time are shown in S2 Fig. Over a 5-year time frame, all values of breeding success led to 100% persistence, as long as adult survival remained above 0.5. The range of vital rate values that resulted in population persistence narrowed as the projected time frame increased to 20 years, and narrowed even further when the time frame increased to 200 years. Over 200 years, the light-colored plateau (S2 Fig) of certain population persistence is very narrow and drops off sharply as adult survival drops below 0.8 and breeding success below 0.5; most predictions below these values are of certain extinction.

The varied vital rates did not have a disproportionate effect on population growth rate in the conventional sensitivity analysis; none of the relative sensitivity values was > 1 or < -1 (Table 3). Juvenile mortality had the highest sensitivity, followed by percent females breeding, adult mortality, breeding success, and environmental variation in adult mortality. Environmental variation in juvenile mortality, carrying capacity, and environmental variation in percent females breeding were the least important. Similarly, logistic regression analysis revealed that juvenile mortality accounted for the most variability in extinction probability, followed closely in importance by adult mortality (Table 3). The third and fourth most important parameters, respectively, were environmental variation in adult mortality and breeding success. Like the conventional sensitivity analysis, carrying capacity and environmental variation in percent females breeding were relatively unimportant to variation in extinction probability. When parameters were modeled singly, regression coefficients indicated a positive relationship between population extinction and each of the following: adult mortality (b = 0.24), juvenile mortality (b = 0.13), and environmental variation in adult mortality (b = 0.10). The remaining variables explained less of the variability in extinction probability (proportion of females with zero broods, i.e. breeding success, b = 0.05; environmental variation in juvenile mortality; b = 0.03; proportion of one-fledgling clutches, b = 0.02; environmental variation in percent females breeding (b = 0.02); carrying capacity, b = -0.00008).

**Table 3:** Sensitivity of population growth and persistence to changes in White-breasted Thrasher parameter estimates.

Parameter	Sensitivity to λ^a^	Sensitivity to PE^b^
*Reproduction*		
Breeding success	0.075 (4)	28.35 (4)
Distribution of fledglings per successful clutch	-	15.58 (5)
Percent females breeding	0.169 (2)	
Environmental variation in percent females breeding	0 (8)	1.27 (8)
*Mortality*		
Juvenile mortality	-0.224 (1)	45.65 (1)
Environmental variation in juvenile mortality	-0.005 (6)	14.28 (6)
Adult mortality	-0.105 (3)	40.31 (2)
Environmental variation in adult mortality	-0.020 (5)	29.94 (3)
*Extrinsic factors*		
Carrying capacity	0.005 (7)	-9.83 (7)

Sensitivity ranks on absolute values are shown parenthetically.

^a^Relative sensitivity to λ was evaluated by varying each parameter ± 10% of its baseline value; see text or Table 1 for baseline values. Negative values indicate a negative relationship between the parameter and population growth.

^b^Sensitivity to PE (probability of extinction) was evaluated by logistic regression standardized coefficients. Negative values do not indicate the relationship between the parameter and population extinction.

### Effects of habitat area

**Fig 3:**
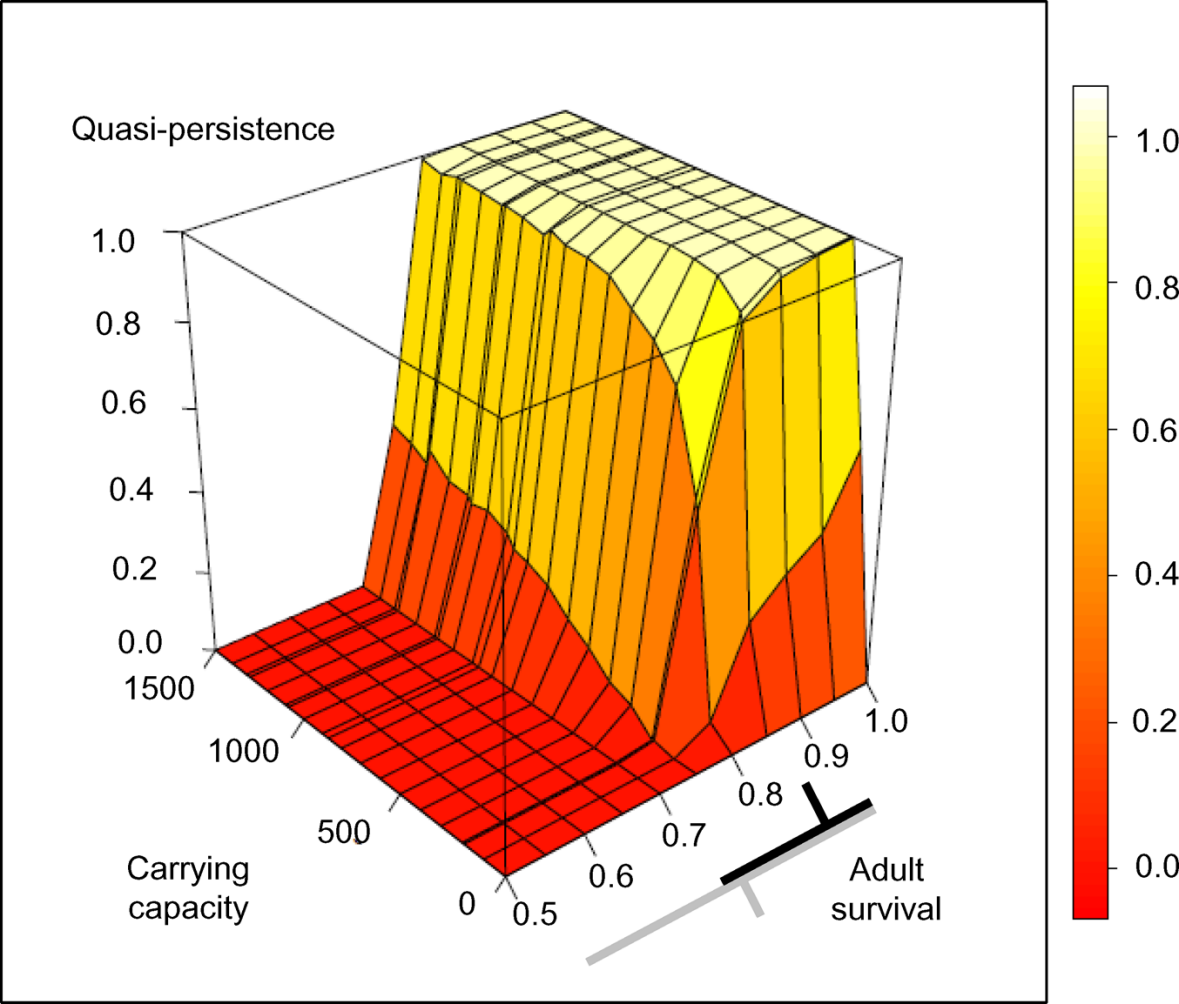
Effects of carrying capacity and annual adult survival on White-breasted Thrasher quasi-persistence over 200 years. We varied carrying capacity from 0–1500 in increments of 100, and also depict the four habitat loss scenarios discussed in the text–from left to right, 1.2K, 1.0K (extant), 0.8K, and 0.17K. The bars on the x-axis represent the mean and 95% CI of adult survival before (black bar) and after (gray bar) habitat loss. Adult survival is truncated below 0.5 because all simulations at lower survival values resulted in a quasi-extinction (defined as a population size < 100) of 1. Note that while simulations were run using adult mortality, survival (1-mortality) is shown here for ease of viewing.

When we varied carrying capacity and adult mortality together, we found a 95% probability of quasi-extinction within 200 years when adult survival fell below 0.8 and carrying capacity below 700 individuals (Fig 3). Quasi-extinction was low across all four discrete habitat area scenarios, as long as adult mortality remained less than or equal to the mean value estimated before habitat loss (as shown by the black bar, Fig 3). Conversely, quasi-extinction was high across all four discrete habitat area scenarios when populations were modeled using adult mortality values similar to those estimated after habitat loss (as show by the gray bar, Fig 3). Across the four specific habitat area scenarios, mean final population size tracked reductions in carrying capacity (S1 Fig); population size at 200 years declined by a mean of 18% with habitat loss between 1.22K and 1K, by 21% between 1K and 0.8K, and a further 78% between 0.8K and 0.17K. For this last scenario, mean final population size was fewer than 165 individuals (S1 Fig). Using occupancy data [67], we found that thrashers occupied 70.8% of the sampling extent within the Mandelé range before habitat loss (corresponding to 1.22K scenario) and 62.1% after habitat loss (corresponding to 1K scenario). Using Eq 2, we calculated an 18.1% reduction in population size between the two periods.

### Population viability analyses of avian cooperative breeders

We found 10 PVAs of seven cooperatively breeding species for which sensitivity analysis was performed in concert with population viability modeling [12, 58, 68–75]; some studies included multiple sensitivity analyses. One additional study reported sensitivity results without performing a PVA [76], and we included two sensitivity analyses from this study, yielding 14 sensitivity analyses of a total of 7 species. The model type and sensitivity method varied greatly between studies, and, interestingly, vital rate sensitivity differed by model type (Table 4). Most studies used matrix models and assessed sensitivity with lower-level sensitivity or elasticity values. Within this group, model structure ranged from 2-stages (e.g., juvenile and adult; [73]) to 6-stages (e.g., juvenile, helper, floater, unpaired territory holder, novice breeder, and experienced breeder; [70]). If we collapse these demographic matrix models to two stages (juvenile and adult), population growth rate was most sensitive to adult survival for most matrix models analyzed. The outliers are the Red-cockaded Woodpecker (*Picoides borealis*) studies, where juvenile survival was the most sensitive vital rate in one analysis, and reproduction the most sensitive vital rate in two other analyses. A fourth Red-cockaded Woodpecker study, with a sensitivity analysis based on a stochastic individual based model, also reported that reproduction most affects extinction risk. In contrast, consistency of sensitivity results within species was seen for the Florida Scrub-Jay and Micronesian Kingfisher (*Todiramphus cinnamominus*); adult survival was the most sensitive parameter across studies for both of these species. In comparison to the matrix models, results for four of the five species modeled with individual based methods, juvenile survival or reproduction was at least as important to population persistence as was adult survival.

**Table 4:** Summary of published sensitivity analyses of avian cooperative breeder vital rates relative to different metrics of population persistence.

								Most sensitive vital rate^h^			
Species	Breeding system^a^	Model type^b^	CB^c^	Method^d^	DV^e^	Metric^f^	Effect size^g^	Juvenile survival	Adult survival	Reprod-uction	Source
Red-cockaded Woodpecker *Picoides borealis*	1	DM^2^	no	1	λ	e	0.59	x			[73]
*P*. *borealis*	1	DM^6^	yes	2	λ	e	0.14			x	[70]
*P*. *borealis*	1	SI	yes	3* (± 10%)	λ	s_x_	0.41			x	[72]
*P*. *borealis*	1	SM^2^	no	4 (+ 1%)	ΔN	ΔN ranked	NA			x	[76]
*P*. *borealis*	1	SM^4^	no	4 (+ 1%)	ΔN	ΔN ranked	NA		x		[76]
Micronesian Kingfisher *Todiramphus cinnamominus*	2	DM^3^	yes	5 (+ 1%)	λ	e	0.48		x		[71]
*T*. *cinnamominus*	2	DM^3^	yes	6	λ	m	0.97		x		[71]
Florida Scrub-Jay *Aphelocoma coerulescens*	2	DM^5^	yes	7	λ	NR	NR		x		[75]
*A*. *coerulescens*	2	SM^6^	yes	3 (± 10%)	qPE	s_x_	0.60		x		[68]
*A*. *coerulescens*	2	SM^1^	yes	3 (± 10%)	qPE	s_x_	0.02	NC	x	xx	[68]
White-banded Tanager *Neothraupis fasciata*	2	SI	no	3*(± 5, 10%)	PE	Δ PE	0.06		x	xx	[12]
Lord Howe Woodhen *Gallirallus sylvestris*	2	SI	no	3* (varies)	PE	s_x_	0.56	x			[69]
Brown Treecreeper *Climacteris picumnus*	1	SI	yes	3* (± 25%)	λ	s_x_	0.04	xx		x	[58]
Helmeted Honeyeater *Lichenostomus melanops cassidix*	2	SI	no	6	PE	b	0.18	NC	x		[74]
White-breasted Thrasher *Ramphocinclus brachyurus*	2	SI	yes	3* (± 10%)	λ	s_x_	0.25	x			present study
*R*. *brachyurus*	2	SI	yes	6*	PE	b	0.12	x			present study

^a^ Type of social breeding system [77]: 1, pair nesting with related male helpers; 2, pair nesting with related male and female helpers.

^b^ Type of model on which sensitivity analysis was performed: DM, deterministic matrix model; SM, stochastic matrix model; SI, stochastic individual-based model. The superscript denotes number of stages in the matrix model.

^c^ Whether cooperative breeding (CB) system was included explicitly in the model.

^d^ Type of sensitivity analysis: 1, eigenvalue sensitivity [78]; 2, eigenvalue elasticity [79]; 3, conventional sensitivity [56]; 4, vital rate sensitivity analysis [76]; 5, brute-force elasticity [80]; 6, logistic regression [59]; 7, method not reported. * Sensitivity analysis was performed on juvenile and adult mortality, rather than survival, for the species denoted. Perturbations to vital rates are noted parenthetically where relevant.

^e^ Dependent variable (i.e. model output) used in sensitivity analysis: λ, per capita geometric rate of increase; N, population size; PE, probability of extinction; qPE, probability of quasiextinction.

^f^ Metric used to evaluate effect of input parameters (P) on dependent variable (x): s_x_, sensitivity index ((Δx/x)/(ΔP/P)); e, elasticity; NR, not reported; m, regression slope; b, scaled regression coefficient. S_x_ for Lord Howe Woodhen calculated by hand from the sensitivity values reported in the manuscript.

^g^ Proportional change in metric between the top two most sensitive parameters. NR, not reported.

^h^ Life-history stages not considered in the model or in sensitivity analysis are indicated by NC. Parameters within an effect size of 10% of the top parameter are indicated by xx. Reproductive parameters used in sensitivity analysis vary by study, but generally include fecundity, variation in fecundity, recruitment, number of nesting attempts yr^-1^, and proportion of individuals breeding.

## Discussion

Our goals were to develop the first demographic, population projection model for the endangered White-breasted Thrasher, to determine model sensitivity to changes in vital rates and carrying capacity, and to compare our sensitivity results to those reported for other avian cooperative breeders. Our models and parameter estimates should be viewed as foundational–our parameters values are based on fewer than eight years of data–and should be validated and updated as additional data become available [10]. Furthermore, our selection of a 200-year time frame for evaluating viability should not be confused with the time frame for management planning. Most management plans are on the order of 5 years; the implicit reason for running a model for more years is that the actual trajectory of a population with stochastic dynamics cannot be revealed adequately in the short term [45–46]. Running a viability model for 5 years, just over a generation for the White-breasted Thrasher, would almost always conclude ‘viability’ even if the population were declining sharply [46]. In addition, if populations can last 40 generations with a high probability, then they can last for five years with a much higher probability. However, errors and uncertainties in parameter values are compounded the longer into the future a model is run. Because of this, we report results for shorter and longer time periods, and have tried to emphasize the relative rather than absolute predictions, as the former are less prone to uncertainty in model structure and parameter estimates [81].

### White-breasted Thrasher individual-based model

Under extant habitat area and pre-habitat loss mortality estimates [34] the Mandelé population is predicted to have a small positive growth rate and low probability of extinction. Other published mortality estimates for the species are available, however, and they do not lead to the same conclusions; using post-habitat loss mortality point estimates [33] in projections results in 100% probability of population quasi-extinction. Our results, however, suggest that these post-habitat loss parameter estimates are pessimistic, at least as a median value. First, unlike the parameter values used in the baseline model [34], there is large uncertainty in the post-habitat loss estimates (juvenile survival 95% CI 0.11–0.64; adult survival 95% CI 0.34–0.94) [33]. The low point estimates and wide confidence intervals were attributed to difficulties in parameter estimation due to low encounter probabilities of banded individuals immediately after habitat loss [33]. Second, if the Mandelé population was experiencing a decline of the magnitude predicted by the high mortality model, we would expect to have detected this decline during fieldwork. If we use the 2007 Mandelé population estimate of 1034 adults [36] and project this value over six years at the mean instantaneous growth rate under high-mortality baseline conditions (N_t_ = N_o_e^rt^), we calculate a predicted population size of 400 adults in 2013. In 2013, > 100 adults were monitored in about 6.5% of the Mandelé range alone (Mortensen unpub. data), and although the population is not homogeneously distributed in Mandelé, much of it is found outside of this small study area [36, 67]. Third, the most recent distance sampling estimate of the entire Mandelé population is over three times higher than that predicted if survival rates were persistently so low [37]. These alternate sources of population estimates suggest that White-breasted Thrasher mortality rates are not as high, or not persistently as high, as estimated immediately following habitat loss. However, mortality rates may not be as low as those simulated under the optimistic pre-habitat loss conditions. The ‘optimistic’ adult White-breasted Thrasher apparent adult annual survival rate estimate of 87% (95% CI: 73–94%; [34]) is higher than that of many other Neotropical birds (e.g., n = 9 species, apparent survival = 0.51–0.78, [82]; n = 17 species, apparent survival = 0.45–0.85, [83]; n = 6 species, apparent survival = 0.30–0.77; [84]), though avian cooperative breeders, such as the White-breasted Thrasher, do tend to have higher annual survival than do non-cooperative breeders [85]. The bounds set by these two mortality estimates–one leads to predictions of population persistence and one to extinction–call attention to the need for additional field estimates of juvenile and adult survival rates. With a robust data set we would also be able to partition sampling and process variance, the former of which should be the discarded and the latter used to incorporate environmental stochasticity into the population projection model [80].

Including behavior in PVAs can improve their predictive ability [16, 23]. For example, [44] used demography and behavior of the cooperatively breeding Red-cockaded Woodpecker to parameterize different types of population models. Of the four candidate model types, the custom-built individual-based spatially explicit model that incorporated key features of the species’ social system produced population size estimates that most closely matched empirical data. In contrast, the two matrix-based models overestimated population size at the final time point, while the individual-based model constructed in VORTEX that did not account for social behavior severely underestimated it, predicting strong population decline. In this case, capturing the dynamics of the breeding system of the modeled species was crucial to accurate model predictions. However, we found that incorporating social behavior into canned viability programs can be challenging, and most VORTEX models of avian cooperative breeders have not explicitly included social behavior. We incorporated aspects of social structure within the confines of VORTEX by constraining the number of breeders in the system to the number of territories, rather than total population size. By modeling White-breasted Thrasher percent breeding as a density-dependent function of number of territories, we attempted to mimic the process of habitat saturation and increasing proportion of nonbreeding helpers as the population grew towards carrying capacity. This treatment is akin to models of non-cooperative species with limited recruitment (e.g., [86]). Explicitly modeling cooperative breeding behavior in our system will require further data collection, e.g., on helper to breeder transition probabilities and helper effect/s on breeder fitness. While there is some evidence that thrasher cooperative groups have higher reproductive output than do lone pairs [34], the generalization of this pattern is unknown, as is whether it is driven by breeder quality, habitat quality, or helper contributions. The potential of increased fitness of certain members of cooperative groups, which is seen in some systems, may be an important parameter to improve PVAs of cooperative species.

Choice of population growth model can influence demographic model output, sometimes leading to qualitatively different conclusions about population dynamics [87]. In PVAs of the Devils Hole Pupfish (*Cyprinodon diabolis*), for example, logistic growth models were used to estimate a median time to extinction of ~26 years [88], while an exponential growth model with a ceiling projected median times to extinction of 1–2 orders of magnitude longer [89]; note that the PVAs differed in other aspects as well, but were based on the same data time series. In our simulations we expected to see highest growth rates for the ceiling model and lowest rates for the VORTEX logistic model, which had the weakest and strongest density-dependence, respectively. Our results fit these predictions; for the scenarios that persisted, the mean stochastic growth rate under the ceiling model was 15% higher than baseline, and under the logistic model it was 48% lower than baseline. Qualitatively, however, our results do not depend on our assumptions about the type and strength of density dependence.

A population viability model is a simplification of a complex system. In our simplification of the White-breasted Thrasher system, we included minimal assessment of animal movements, a variable known to affect population persistence for some species (e.g., [90]). Disrupted dispersal can impact population persistence by eliminating rescue if the population begins to decline [91], and this effect was reported from demographic models of other avian cooperative breeders (e.g., [19, 92]). We found that dispersal into Mandelé had little effect on population dynamics beyond marginally extending time to extinction in the high-mortality scenarios. We did not model movements within Mandelé, but expect that movements are largely uninterrupted, as the range still has a relatively high extent of habitat [93], thrashers are willing to cross small gaps within fragmented areas (Mortensen pers. obs.), and in parts of the range, territories are highly aggregated [33]. Furthermore, we do not know if Mandelé–the stronghold of the species–is contributing to the other populations. Including dispersal information in viability modeling may be more important for understanding and predicting extinction risk of the Saint Lucian Iyanola population, which is much smaller than Mandelé and has declined over the last 40 years [36].

### Model sensitivity

Sensitivity analysis can be used to quantify the extent to which changes in vital rates affect the population trajectory of a species of interest [10]. To be of use in species management, the outcome of a sensitivity analysis must be interpreted in light of the capacity for vital rates to change, i.e. the historic variation of the key vital rate and the scope for management to improve it [24]. We found that by systematically changing each of our parameters of interest–successful breeding attempts, juvenile mortality, and adult mortality–one at a time, we could cause relatively sudden increased extinction probability and decreased population size. Of the three vital rates, adult mortality had the most severe threshold and the least difference between empirical estimate and threshold value. Logistic regression, where multiple parameters were varied simultaneously, also identified adult mortality as highly sensitive in that it was important in explaining variability in extinction probability. However, this vital rate may have the least capacity to change compared to the other vital rates because it is already relatively low. In fact, less capacity for change is an expectation of the canalization hypothesis, where key parameters are constrained by natural selection to have low variability [94]. In contrast, juvenile mortality had the largest relative sensitivity in its ability to affect population growth rate as well as probability of extinction, and because the mean estimate is so high, it may be the rate with the most capacity for improvement. As the primary cause(s) of juvenile mortality are not known, to understand the capacity of management to affect this vital rate will require further study.

### Effects of habitat area

Habitat loss is the primary deterministic threat to bird species globally [95]. It is one of the justifications for the White-breasted Thrasher’s endangered status on the IUCN Red List [35], and subsequently, stemming habitat loss is one of the three main objectives in the recent conservation plan for the Saint Lucian subspecies [37]. Our analyses suggest that there is currently sufficient habitat to ensure White-breasted Thrasher persistence in the Mandelé range for the near future, as long as mortality levels remain low, close to the values estimated before habitat loss. In the simulation, recent habitat loss (a shift from 1.2 to 1.0K) did little to affect population persistence measures, but resulted in an average 18% decline in projected population size, relative to the population simulation under conditions without the loss. Interestingly, using a method to predict population change using occupancy data [66], we also found an 18% predicted reduction in population size associated with habitat loss. This was after adjusting for the change in group size caused by the cooperative breeding social system; thrasher family group size increased when habitat was lost [33], while in a monogamous species this would not be expected, and greater population decline may occur. To our knowledge, ours is the first field test of [66]. The similarity between predictions suggests that this simple occupancy-based method should be further investigated with field data, and where appropriate, corrected for the density-abundance relationship associated with social behavior.

Since 2005, the Mandelé range has lost about 20% of White-breasted Thrasher habitat in association with resort construction and further loss is expected [37]. How this anticipated future loss affected population persistence in the long term in our simulation depended on annual mortality. Within the range of ‘optimistic’ adult mortality values (i.e. 95% CI of pre-habitat loss estimate), the risk of quasi-extinction tended to accelerate as carrying capacity fell below 700–800 individuals. 700–800 individuals, under our estimates of White-breasted Thrasher density, is slightly less than the habitat area expected to remain in the Mandelé range after resort construction is complete [37]. This result should not be taken as an estimate of minimum viable population size (MVPS) on which to base management planning for this species. Researchers have repeatedly warned against taking a ‘minimum’ approach to species conservation planning (e.g., [24, 96]). Our vital rate estimates are made from a fairly short time series of data, and [97] showed that as the length of a study increased, the higher the estimate of MVPS because longer studies do a better job of capturing environmental stochasticity. In addition, [98] used empirical and theoretical data to estimate targeting minimum management goals for vertebrates of thousands of individuals to ensure long-term persistence (see also [64]). Consequently, to set a confident target management goal below this size would require strong supporting evidence from long-term studies.

### Vital rate sensitivity of avian cooperative breeders

Reviews of vital rate sensitivity in birds often report adult survival as the most sensitive vital rate in population models [99, 100]. This pattern, however, is far from universal. In a review of 155 populations of 113 species, adult survival had the largest influence on growth rate in only 53.5% of the analyses [101]. In another review of 23 species, juvenile survival had the largest contribution to growth rate for 80% of the species [102]. In general, and perhaps not surprisingly, they noted, as did [100], that fecundity or juvenile survival was more sensitive for species with high fecundity, and adult survival was more important in long-lived species. Our review of avian cooperative breeders resulted in a similar mix of results. We found adult survival to be the vital rate identified as the most sensitive for half of the avian cooperative breeders. Of the 16 sensitivity analyses we evaluated (including our study), eight (50%) showed this pattern. In two of these analyses, however, reproduction was nearly as sensitive as was adult survival. For all four species with multiple sensitivity analyses, there was inconsistency among intra-specific analyses, but for only one of these species, inconsistencies resulted in different vital rates being identified as most sensitive. Specifically, in the five analyses of Red-cockaded Woodpecker, four sensitivity methods gave rise to each of three vital rates being identified as most sensitive. Besides use of different sensitivity methods, these intra-specific models differ in their matrix dimensions, which has been shown to influence elasticity estimates in plants [103]. In general, sensitivity results were split among type of sensitivity analysis–adult survival was most important in studies that used matrix models and analytical elasticity methods, which most often assessed sensitivity to population growth rate, and less important in individual-based modeling studies, which tended to assess sensitivity to probability of extinction. Interestingly, these two types of models were also split in their use of survival vs. mortality rates, i.e. all matrix models projected survival and individual-based models tended to project mortality. This use of vital rates in different ways makes comparison between studies difficult [80]. Besides the types of models and sensitivity analyses run, life-history causes, such as evolutionary covariates of generation length, may contribute to the variation seen in sensitivity analyses, as proposed by [101]. However, in our set of avian cooperative breeders, we see no apparent pattern in average longevity and which parameter is most sensitive (S1 Table). It remains to be seen whether the differences we found in sensitivity results for cooperative breeders are due to biological or analytical differences. Either way, clarification is of interest and concern for species management.

Because White-breasted Thrashers have low reproductive capacity (2-egg clutch, generally ≤ 2 successful clutches per year; [33, 34]), we anticipated that adult mortality would be the most sensitive vital rate [102]. While this vital rate was important in that it had observed values closest to the threshold above which populations would crash, both population growth and population persistence were most sensitive to juvenile mortality. It has been suggested that cooperative breeding, particularly the capacity to fill empty breeder slots by helpers (e.g., [27, 29]), might result in variation in reproduction or juvenile mortality being the most sensitive vital rate [12]. But, again, this was not typical for cooperative breeders. Determining if there is a mechanism via social behavior that biases population growth sensitivity towards a particular vital rate is an interesting challenge that will require behavior-specific components of population models. Of the four models from Table 4 that explicitly included helper to breeder transitions, three found highest vital rate sensitivity for reproduction parameters [58, 70, 72] whereas the other one found highest sensitivity for adult survival [71]. Further viability modeling of cooperative systems will contribute to understanding the dynamics and extinction risk of species with this unusual breeding system.

## Supporting Information

S1 Fig Effects of mortality level and carrying capacity on White-breasted Thrasher population size and time to extinction. Carrying capacity models correspond to past (1.2K), present (1K), and proposed future (0.8K and 0.17K) amounts of habitat available in the Mandelé range. All scenarios were run under baseline conditions (other than the varied carrying capacity level) over a 200-year time frame. The upper and lower box edges correspond to the 25^th^ and 75^th^ percentiles, whereas the whiskers extend to the highest and lowest values within 1.5*IQR (inter-quartile range). Data beyond the whiskers are plotted as points.
(TIF)

S2 Fig Effects of co-varying White-breasted Thrasher breeding success and adult survival on population persistence at 5, 20, and 200-year time frames under baseline conditions. Breeding success is the proportion of breeding females that have at least one successful brood per season. Note that simulations were run using adult mortality; survival (1-mortality) is shown here for ease of viewing.
(TIF)

S1 Table Vital rate sensitivity in relation to longevity of avian cooperative breeders. (DOC)
