# Supplementary figures and images for: Population Viability and Vital Rate Sensitivity of an Endangered Avian Cooperative Breeder, the White-Breasted Thrasher (Ramphocinclus brachyurus)
Source: PLoS One. 2016 Feb 9;11(2):e0148928. doi: 10.1371/journal.pone.0148928 (PMC4747538; doi:10.1371/journal.pone.0148928)

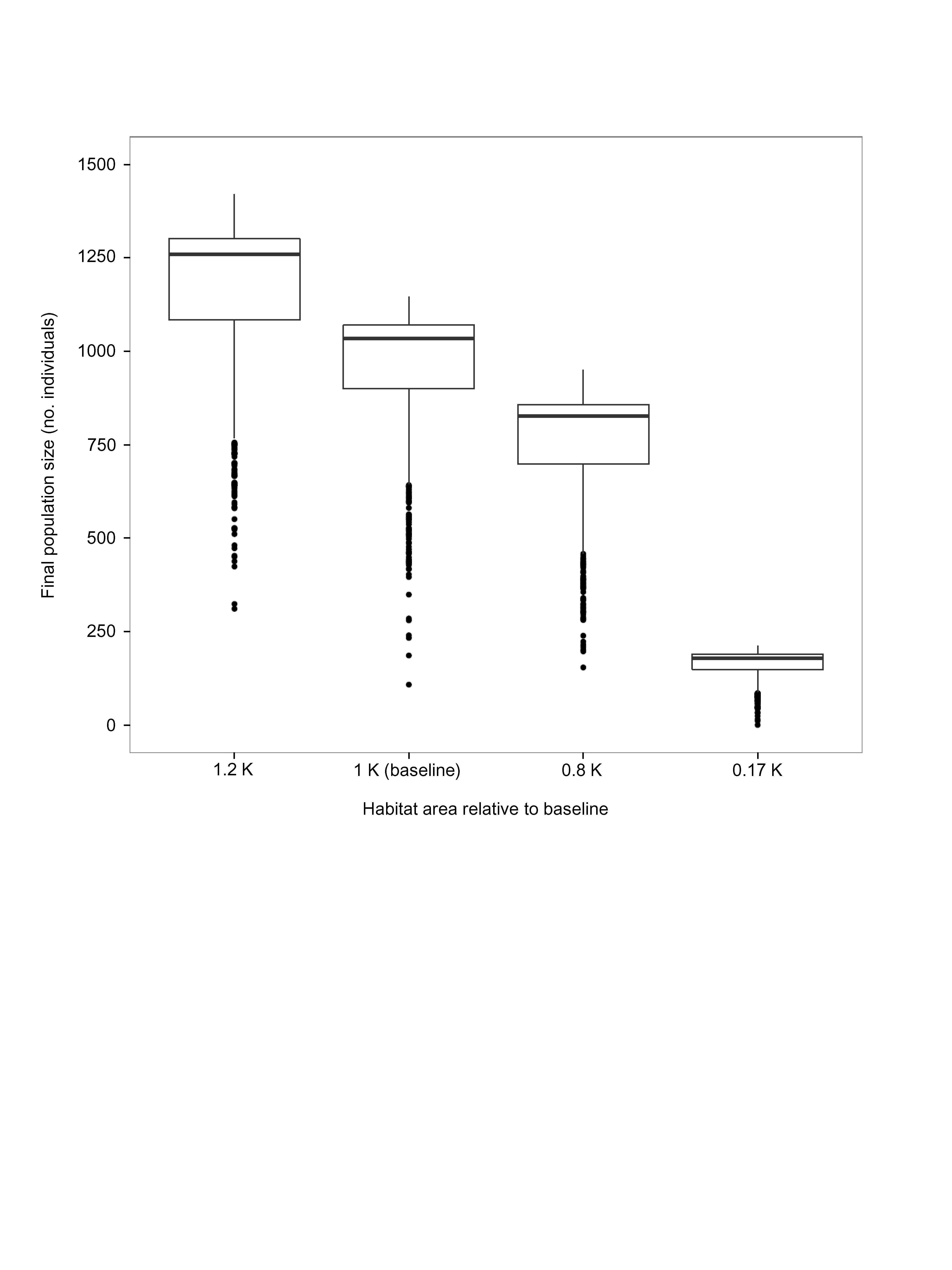

Supplement: S1 Fig — Carrying capacity models correspond to past (1.2K), present (1K), and proposed future (0.8K and 0.17K) amounts of habitat available in the Mandelé range. All scenarios were run under baseline conditions (other than the varied carrying capacity level) over a 200-year time frame. The upper and lower box edges correspond to the 25th and 75th percentiles, whereas the whiskers extend to the highest and lowest values within 1.5*IQR (inter-quartile range). Data beyond the whiskers are plotted as points. (TIF) [file pone.0148928.s001.tif]

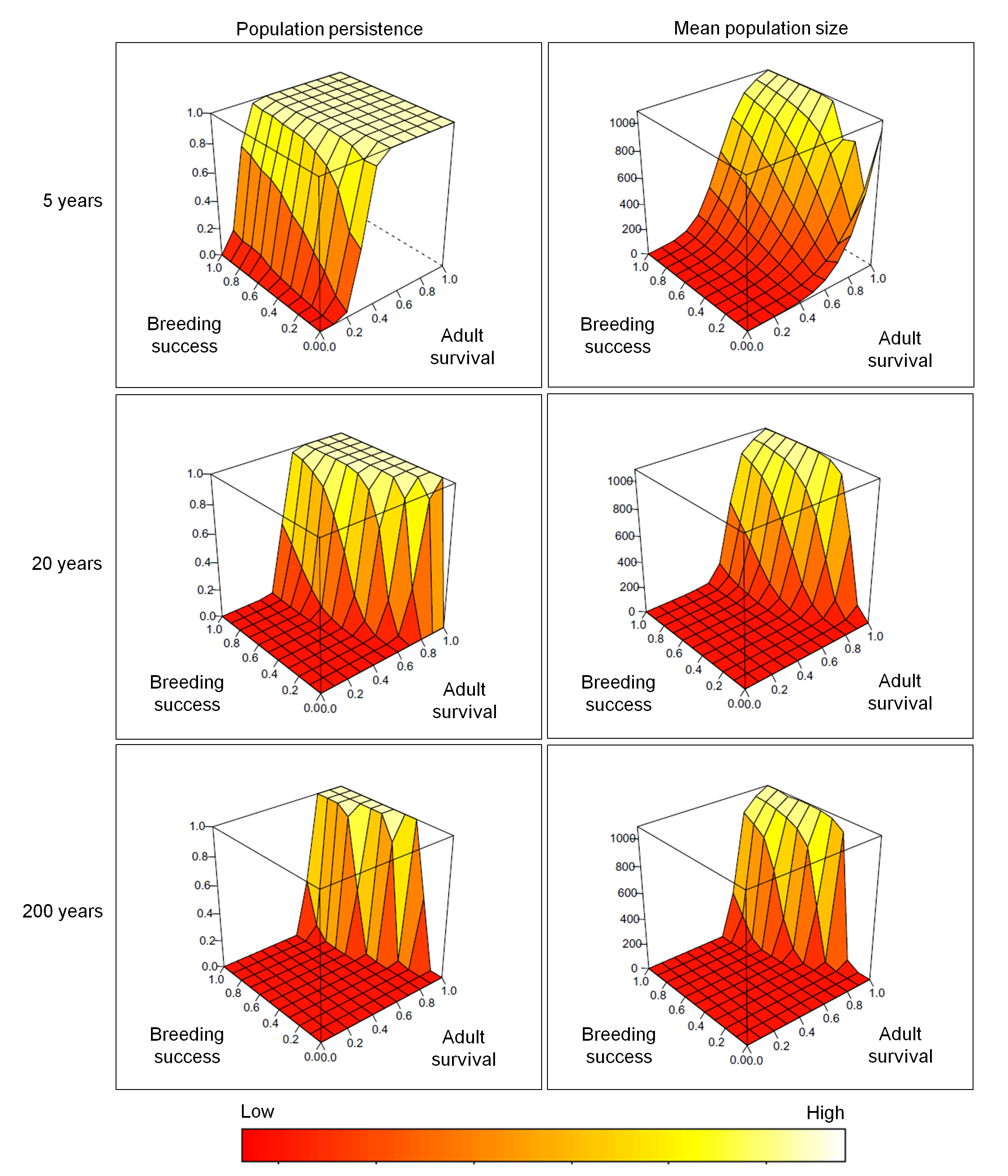

Supplement: S2 Fig — Breeding success is the proportion of breeding females that have at least one successful brood per season. Note that simulations were run using adult mortality; survival (1-mortality) is shown here for ease of viewing. (TIF) [file pone.0148928.s002.tif]
