# Supplementary material for: Population Viability and Vital Rate Sensitivity of an Endangered Avian Cooperative Breeder, the White-Breasted Thrasher (Ramphocinclus brachyurus)
Source: PLoS One. 2016 Feb 9;11(2):e0148928. doi: 10.1371/journal.pone.0148928 (PMC4747538; doi:10.1371/journal.pone.0148928)
Supplement: S1 Table — (DOC) [file pone.0148928.s003.doc]

S1 Table. Vital rate sensitivity in relation to longevity of avian cooperative breeders.

|  | Longevity^a^ | Most sensitive^b^ | | | Citation if >1^c^ |
| --- | --- | --- | --- | --- | --- |
|  |  | Juvenile | Adult | RS |  |
| Micronesian Kingfisher  *Todiramphus cinnamominus* | 1/0.423 = 2.36 |  | 2 |  |  |
| Lord Howe Woodhen  *Gallirallus sylvestris* | 1/0.32 = 3.13 | 1 |  | 1 |  |
| White-banded Tanager *Neothraupis fasciata* | 1/0.266 = 3.76 |  | 1 |  |  |
| Helmeted Honeyeater *Lichenostomus melanops*  *cassidix* | 1/0.235 = 4.26 |  | 1 |  |  |
| Brown Treecreeper *Climacteris picumnus* | 1/0.23 = 4.35 | 1 |  | 1 |  |
| Red-cockaded Woodpecker *Picoides borealis* | 1/0.23 = 4.35 | 1 | 1 | 3 | [72] |
| Florida Scrub-Jay *Aphelocoma coerulescens* | 1/0.19 = 5.26 |  | 3 |  | [75]; averaged values for breeders w/ & w/o helpers |
| White-breasted Thrasher *Ramphocinclus brachyurus* | 1/0.13 = 7.69 | 2 |  |  |  |

^a^ There is a reciprocal relationship between death rate and longevity (e.g., Sandercock 2003). Average longevity (average age at death) was estimated as 1/adult mortality rate. Data are from male breeders if there was a choice. Helmeted Honeyeater data are breeding season survival.

[Sandercock, B.K. 2003. Estimation of survival rates for wader populations: a review of mark-recapture methods. Wader Study Group Bulletin 100:163-174.]

^b^ The number here is the number of studies that conclude a particular vital rate is most sensitive. RS = reproductive success.

^c^ See text for citations.
